# Combining Clinicopathology, IVIM-DWI and Texture Parameters for a Nomogram to Predict Treatment Response to Neoadjuvant Chemoradiotherapy in Locally Advanced Rectal Cancer Patients

**DOI:** 10.3389/fonc.2022.886101

**Published:** 2022-05-27

**Authors:** Rixin Su, Shusheng Wu, Hao Shen, Yaolin Chen, Jingya Zhu, Yu Zhang, Haodong Jia, Mengge Li, Wenju Chen, Yifu He, Fei Gao

**Affiliations:** ^1^Department of Medical Oncology, Anhui Provincial Hospital Affiliated to Anhui Medical University, Hefei, China; ^2^Department of Medical Oncology, The First Affiliated Hospital of University of Science and Technology of China (USTC), Division of Life Sciences and Medicine, University of Science and Technology of China, Anhui Provincial Cancer Hospital, Hefei, China; ^3^Department of Radiology, The First Affiliated Hospital of University of Science and Technology of China (USTC), Division of Life Sciences and Medicine, University of Science and Technology of China, Anhui Provincial Cancer Hospital, Hefei, China

**Keywords:** locally advanced rectal cancer, clinicopathology, intravoxel incoherent motion diffusion weighted imaging, texture analysis, nomogram, prediction, response to nCRT

## Abstract

**Objectives:**

This study aimed to create a nomogram for the risk prediction of neoadjuvant chemoradiotherapy (nCRT) resistance in locally advanced rectal cancer (LARC).

**Methods:**

Clinical data in this retrospective study were collected from a total of 135 LARC patients admitted to our hospital from June 2016 to December 2020. After screening by inclusion and exclusion criteria, 62 patients were included in the study. Texture analysis (TA) was performed on T2WI and DWI images. Patients were divided into response group (CR+PR) and no-response group (SD+PD) according to efficacy assessment. Multivariate analysis was performed on clinicopathology, IVIM-DWI and texture parameters for screening of independent predictors. A nomogram was created and model fit and clinical net benefit were assessed.

**Results:**

Multivariate analysis of clinicopathology parameters showed that the differentiation and T stage were independent predictors (OR values were 14.516 and 11.589, resp.; *P*<0.05). Multivariate analysis of IVIM-DWI and texture parameters showed that f value and Rads-score were independent predictors (OR values were 0.855, 2.790, resp.; *P*<0.05). In this study, clinicopathology together with IVIM-DWI and texture parameters showed the best predictive efficacy (AUC=0.979). The nomogram showed good predictive performance and stability in identifying high-risk LARC patients who are resistant to nCRT (C-index=0.979). Decision curve analyses showed that the nomogram had the best clinical net benefit. Ten-fold cross-validation results showed that the average AUC value was 0.967, and the average C-index was 0.966.

**Conclusions:**

The nomogram combining the differentiation, T stage, f value and Rads-score can effectively estimate the risk of nCRT resistance in patients with LARC.

## Introduction

Rectal cancer is the third most common malignancy in terms of morbidity and mortality worldwide, and nearly half of rectal cancers are diagnosed as locally advanced rectal cancer (LARC) (1). Total mesorectal excision (TME) after neoadjuvant chemoradiotherapy (nCRT) for LARC is the current standard treatment modality, which can improve the local control rate, sphincter preservation rate, and reduce the local recurrence rate of LARC. And 10% to 20% of patients can achieve pathological complete remission after nCRT (2). Early assessment of nCRT efficacy and preservation of anal sphincter function can significantly improve the quality of life for patients (3, 4). Therefore, it becomes an urgent issue to find markers that can predict the efficacy of nCRT for LARC, then differentiate patients who are sensitive or resistant to nCRT at an early stage, and formulate individualized treatment for different patients.

Since the introduction of conventional MRI to local staging of rectal cancer, many efforts have been made to find predictive imaging signatures so as to identify patients with good or poor response to nCRT and patients at higher risk of recurrence (5, 6). However, as conventional MRI has poor sensitivity and specificity in assessing and predicting treatment response, it is hard to distinguish post-treatment fibrosis and edema from tumor tissue after nCRT, and even harder to accurately predict complete pathological remission (7–9). Without the use of exogenous contrast agents, intravoxel incoherent motion diffusion weighted imaging (IVIM-DWI) based on the biexponential model can assess the pure diffusion motion and perfusion-related motion of water molecules separately. Compared to conventional MRI, IVIM-DWI can more accurately display tissue microenvironment information and non-invasively and quantitatively diagnose tumor malignancy, pathological differentiation, and lymph node metastasis (10–12). In recent years, several studies have proved the superiority of IVIM-DWI in predicting treatment response in various tumors (13–15). It was found that IVIM-DWI was effective in predicting tumor size changes in patients with breast cancer liver metastases who were undergoing radioembolization, and that treatment-induced changes in f value could be a potential biomarker in the prediction of treatment response (16). Studies have also shown that IVIM-DWI parameters, especially pretreatment D value could predict patients’ response to induction chemotherapy with locally advanced hypopharyngeal carcinoma (17). However, no conclusive results have been obtained about the efficacy of IVIM-DWI in predicting the response of patients with LARC to nCRT.

Texture analysis (TA) is an emerging image analysis method that extracts quantitative imaging features from images through high throughput analysis. It can reflect the spatial variation and heterogeneity of voxel intensities within the tumor and can provide valuable references for disease diagnosis, treatment, and efficacy prediction of therapies (18, 19), thus a promising method for assessing response to various cancer treatments. Studies have found that texture analysis of magnetic resonance imaging (MRI) could predict early recurrence after hepatectomy for solitary hepatocellular carcinoma (20). It was also found that MRI texture features could predict pathological complete response (pCR) to neoadjuvant chemotherapy in breast cancer (21). In rectal cancer, TA can work as a supplement to conventional MR imaging, especially in identifying staging, assessing treatment response, and predicting lymph node metastasis and prognosis (22–24).

The purpose of this study was to combine clinicopathology, IVIM-DWI and texture parameters to establish a nomogram for treatment response prediction to nCRT in patients with LARC, which can be used to identify patients at high risk of nCRT resistance.

## Materials and Methods

### Patients

**Figure 1:**
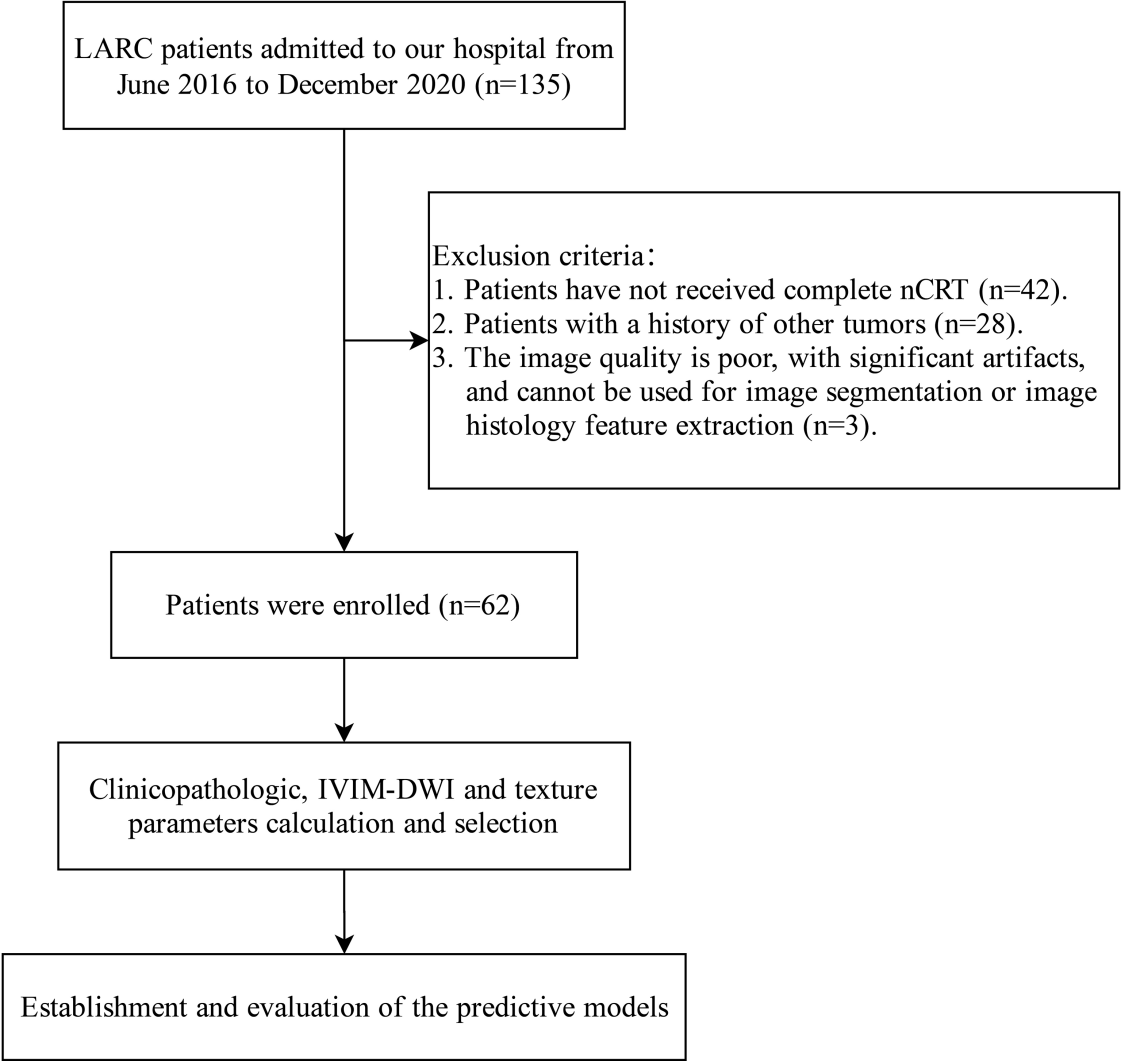
Flow chart for establishing a nomogram for predicting nCRT resistant in LARC patients.

A total of 135 LARC patients admitted to Anhui Provincial Hospital from June 2016 to December 2020 were retrospectively collected, among whom 62 patients with complete clinical data were included in this study. Inclusion criteria included (1) Locally advanced rectal primary adenocarcinoma was confirmed by colonoscopy biopsy; (2) All patients received pelvic nCRT before surgery; (3) The lower edge of the tumor is within 15 cm of the anal verge; (4) Patients completed routine MRI and IVIM-DWI examinations before nCRT. Exclusion criteria were (1) Patients who have not received complete nCRT; (2) Patients with a history of other tumors; (3) The image quality is poor, with significant artifacts, and cannot be used for image segmentation or image histology feature extraction. The flow chart of this study is shown in **Figure 1**.

### Imaging Examination

Before receiving nCRT, patients underwent MRI examination which was performed in the supine position utilizing a 3.0-T scanner (Signa HDXT, General Electric Healthcare, Waukesha, WI, USA) with an 8-channel phased array coil. All patients fasted for at least 8 hours and received intramuscular injection of hyoscine butylbromide 20 mg half an hour before MRI examination to reduce gastrointestinal motility artifacts. The sequences include axial T_1_-weighted imaging (T1WI) fast spin-echo (FSE), axial T_2_-weighted imaging (T2WI) FSE, sagittal T2WI FSE, coronal T2WI FSE. IVIM-DWI was acquired using single-shot echo-planar imaging (EPI) pulse sequence with 10 b values (0, 10, 20, 50, 100, 200, 400, 800, 1,200, and 2000 s/mm^2^). The scan parameters of each sequence in MRI are shown in **Table 1**.

**Table 1:** Scan parameters of each sequence in MRI.

Sequences	T1WI FSE	T2WI FSE	T2WI FSE	T2WI FSE	IVIM-DWI
Plane	Axial	Axial	Sagittal	Coronal	Axial
TR/TE (ms)	500/7.2	3500/109.1	3500/109.1	3500/109.1	4000/75
NEX	1	4	4	4	–
FOV (mm)	320 × 320	240 × 240	240 × 240	240 × 240	420 × 420
Slice thickness (mm)	6	3	3	3	4
Slice interval (mm)	2	0	0	0	1

T1WI, T_1_-weighted imaging; FSE, fast spin-echo; T2WI, T_2_-weighted imaging; TR/TE, repetition time/echo time; NEX, number of excitations; FOV, field of view.

### IVIM-DWI Measurement

**Figure 2:**
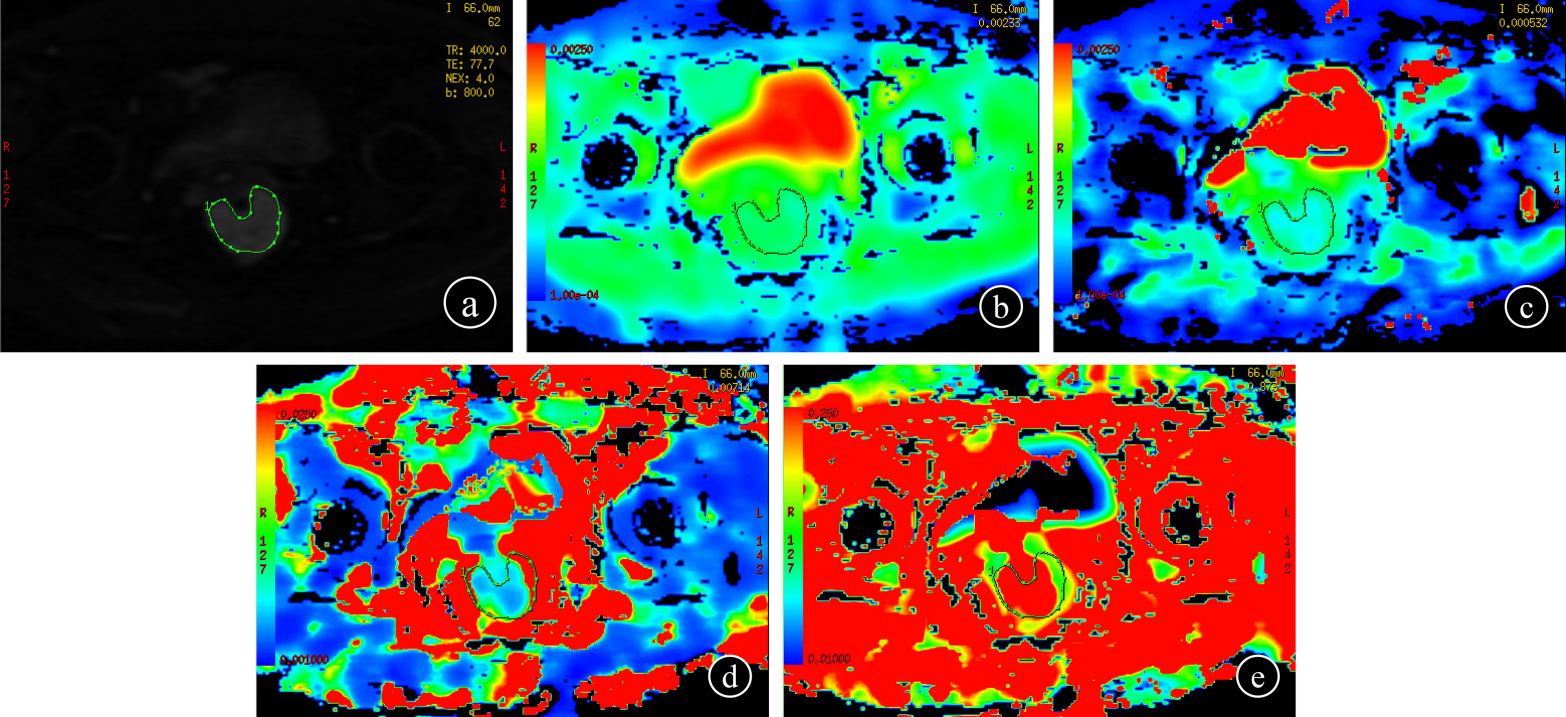
IVIM-DWI measurement (b = 800 s/mm^2^) **(A)**; each radiologist drew ROI three times to get the values on the maps of ADC, D, D^∗^, and f, respectively **(B–E)**.

The IVIM-DWI measurement was performed by two radiologists. They used FunctionTool to build regions of interest (ROI) on a post-processing workstation (version ADW 4.5, GE Healthcare) without knowledge of clinicopathological results. To acquire the parameters, the ROI of IVIM-DWI was selected on the maximum transverse plane of each lesion (b value=800 s/mm^2^) (**Figure 2**). Referring to the axial T2WI image, the ROI was manually delineated along the tumor margin, avoiding the hemorrhage, necrosis, and cystic change of the tumor. All parameters were measured three times and the average value was calculated. To assess interobserver agreement, each radiologist plotted twice to obtain values and calculate intraclass correlation coefficients (ICC). Parameters with ICC value > 0.75 were selected for subsequent analysis.

### Texture Features Analyses

**Figure 3:**
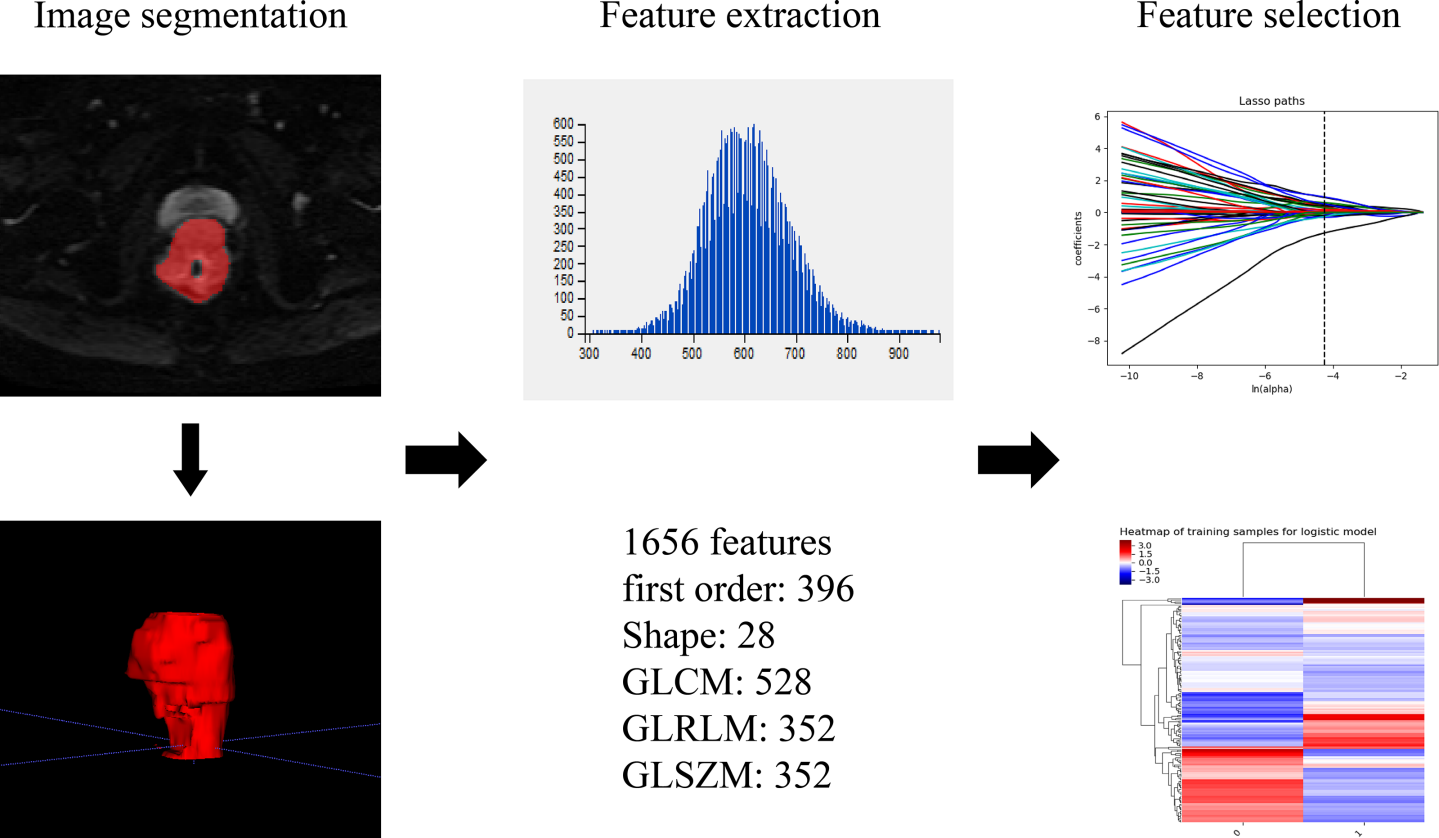
Texture features acquisition workflow.

Patients underwent tumor delineation and texture features extraction. The raw data was transferred to the PACS system, and axis IVIM-DWI (b=800 s/mm^2^) and T_2_WI images (DICOM format) were imported into the ITK-SNAP software. Two radiologists segmented the tumor’s 3-dimensional volume of interest (3D-VOI), and all lesions were delineated layer by layer. The definition of ROI included areas of hemorrhage, necrosis, and cystic degeneration while avoiding normal anatomy. The original images and ROIs were imported into A.K (Analysis Kit, Kinetics Version 2.1, GE Healthcare). Then a series of texture features for all lesions were automatically acquired by the software. Finally, 1 656 texture features (828 on T2WI, 828 on IVIM-DWI) of the entire tumor were extracted. **Figure 3** shows the texture feature extraction. Features with ICC value > 0.75 were selected for subsequent analysis.

### nCRT Regimen

nCRT regimen: radiotherapy (5 days a week, 1.8 ~ 2.0 Gy/d, total dose of 45 ~ 50 Gy) + chemotherapy [capecitabine 825 mg/m^2^, twice a day (Monday to Friday)].

### nCRT Efficacy Evaluation

nCRT efficacy on patients was assessed 6-8 weeks after they received nCRT. Two senior physicians with rich experience in pelvic MR diagnosis performed image evaluation on fat suppressed T2WI (T2WI-FS) sequences under double-blind conditions. They used histology as the reference standard to identify responders or not. The efficacy evaluation was based on the revised RECIST guidelines (version 1.1) criteria (25): (1) Complete response (CR) refers to the disappearance of all target lesions, the appearance of no lesions, and the normal tumor markers for at least 4 weeks. (2) Partial remission (PR) is the reduction of the sum of the maximum diameters of target lesions ≥30% for at least 4 weeks. (3) Stable disease (SD) is the sum of the largest diameters of target lesions and the reduction does not reach PR, or the enlargement does not reach disease progression (PD). (4) PD refers to an increase of 20% at least in the sum of the largest diameters of target lesions, or the appearance of new lesions. Response group = CR+PR, No-Response group = SD+PD.

### Statistical Analysis

All data were statistically analyzed using Graphpad Prism 9.0, SPSS 26.0, R 4.0.5 and IPMS 2.4.0 software. For continuous variables, Kolmogorov-Smirnov was used to test the normality of the data. If the data followed normal distribution, x ± s was used for statistical description, and independent sample t-test was conducted to compare two groups; otherwise, median ± interquartile range was used for statistical description, and Mann-Whitney U test was conducted to compare two groups. For categorical variables, the number and percentage of cases were used for statistical description, and the chi-square test or Fisher’s exact probability was adopted for comparison between two groups. ICC assessed the inter-observer and intra-observer agreement of IVIM-DWI values and texture feature measurements (ICC>0.75 indicated good agreement) (26, 27). Radiomic features were screened by independent samples t-test or Mann-Whitney U test and multivariate logistic regression. Each set of texture features was developed using multi-factor linear weighting based on the regression coefficients of the selected features, and a radiomics score (Rads-score) was calculated for each patient. A nomogram model was established on the basis of clinicopathology, IVIM-DWI and Rads-score parameters. Ten-fold cross-validation was performed to assess discrimination and prediction ability of the nomogram model. All statistical tests were two-sided probability tests, and *P* <0.05 was considered statistically significant.

### Ethical Approval and Informed Consent

This study was approved by the Ethics Committee of Anhui Provincial Hospital and the requirement for informed consent was waived due to the retrospective nature of this study.

## Results

### Clinicopathology Characteristics

A total of 62 patients’ data were collected. The average age of the patients was 58.77 ± 15.66 years. The clinical characteristics of the patients were shown in **Table 2**. All patients completed the efficacy evaluation, including 6 cases of CR (9.68%), 38 cases of PR (61.29%), 18 cases of SD (29.03%), 0 cases of PD (0%); ORR was 70.97% and DCR was 100%. There were no significant differences in age, gender, tumor diameter, N stage, CA199, and HB between the two groups (*P*>0.05).

**Table 2:** Clinicopathologic characteristics in rectal cancer Patients.

Characteristics	Response group (n = 44)	No-Response group (n = 18)	*P* value
Age (year)	59.4 ± 15.5	57.2 ± 16.5	0.609
Sex			0.954
Male	29	12	
Female	15	6	
Differentiation			0.022
Well/Moderate	41	12	
Poor	3	6	
Tumor diameter (cm)	4.4 ± 2.1	5.2 ± 2.2	0.178
T stage			0.003
T2~3	28	4	
T4	16	14	
N stage			0.492
N0	7	1	
N1~2	37	17	
CEA (ng/ml)			0.008
<5	26	4	
≥5	18	14	
CA199 (U/ml)			0.068
<37	36	10	
≥37	8	8	
HB (g/l)	120.5 ± 17.6	119.4 ± 20	0.829

CEA, carcinoembryonic antigen; CA199, carbohydrate antigen 19-9; HB, Hemoglobin; CR, complete response; PR, partial response; SD, stable disease; PD, disease progression; Response group, the first-time evaluation results was CR or PR; No-Response group, the first-time evaluation results was SD or PD.

### Intra- and Inter-Observer Agreement

Among the 1 656 texture features extracted from T2WI and IVIM-DWI (b=800 s/mm^2^) images, there were 1 054 text features with high stability. The ICC (inter) and ICC (intra) of IVIM-DWI values and texture features ranged from 0.775 to 0.996 and from 0.764 to 0.998, respectively, with good repeatability.

### Calculating the Radsscore

Among the texture features extracted from axial T_2_WI and IVIM-DWI (b=800 s/mm^2^), two statistically significant texture feature parameters [glrlm_LongRunEmphasis (T_2_WI), firstorder_Median (IVIM)] were retained for diagnostic efficacy after feature selection analysis. The Rads-score formula of the radiomics is as follows:


$$\text{Rads}-\text{score}=-6.627+0.269*\text{glrlm}\_\text{LongRunEmphasis}\left({\text{T}}_{2}\text{WI}\right)+0.003*\text{firstorder}\_\text{Median}\left(\text{IVIM}\right)$$

### Predictive Performance of Clinicopathology Parameters

Univariate analysis showed that tumor differentiation, T stage, CEA and CA199 were significantly correlated with the efficacy of nCRT in patients (*P*<0.05). Multivariate logistic analysis showed that tumor differentiation (OR: 14.516, 95%CI: 1.726-122.057, *P*=0.014) and T stage (OR: 11.589, 95%CI: 2.103-63.860, *P*=0.005) were independent factors affecting nCRT efficacy (**Table 3**). Therefore, a predictive model (model 1) was developed.

**Table 3:** Statistical analysis results of clinicopathologic characteristics in rectal cancer patients.

Characteristics	Univariate analysis (*P*)	Multivariate logistic regression analysis		
		OR	95%CI	*P*
Age (year)	0.603			
Sex	0.954			
Differentiation	0.014	14.516	1.726-122.057	0.014*
Tumor diameter (cm)	0.179			
T stage	0.005	11.589	2.103-63.860	0.005*
N stage	0.292			
CEA (ng/ml)	0.012	3.825	0.764-19.142	0.103
CA199 (U/ml)	0.037	2.464	0.460-13.208	0.292
HB (g/l)	0.825			

*P < 0.05.

CEA, carcinoembryonic antigen; CA199, carbohydrate antigen 19-9; HB, Hemoglobin; CR, complete response; PR, partial response; SD, stable disease; PD, disease progression; Response group, the first-time evaluation results was CR or PR; No-Response group, the first-time evaluation results was SD or PD.

### Predictive Performance of IVIM-DWI and Texture Parameters

Univariate analysis showed that D, D*, f and Rads-score were significantly correlated with the efficacy of nCRT in patients (*P*<0.05). Multivariate logistic analysis showed that f (OR: 0.855, 95%CI: 0.752-0.971, *P*=0.016) and Rads-score (OR: 2.790, 95%CI: 1.163-6.696, *P*=0.022) were independent factors influencing nCRT efficacy in patients (**Table 4**). Therefore, a predictive model (model 2) was built to describe the relations between independent factors and nCRT efficacy.

**Table 4:** Statistical analysis results of the IVIM-DWI and texture parameters in rectal cancer patients.

Parameters	Response group	No-Response group	Univariate analysis (*P*)	Multivariate logistic regression analysis		
				OR	95%CI	*P*
ADC (×10^−3^ mm^2^/s)	0.935 ± 0.256	0.996 ± 0.193	0.364			
D (×10^−3^ mm^2^/s)	0.593 ± 0.194	0.846 ± 0.308	0.002	2.717	0.019-380.669	0.692
D∗(×10^−3^ mm^2^/s)	14.62 ± 13.73	24.74 ± 20.05	0.038	0.976	0.906-1.052	0.530
f (%)	47.01 ± 13.95	27.03 ± 10.33	<0.001	0.855	0.752-0.971	0.016*
Rads-score	4.67 ± 1.19	6.14 ± 1.31	0.002	2.790	1.163-6.696	0.022*

*P < 0.05.

IVIM-DWI, intravoxel incoherent motion diffusion weighted imaging; CR, complete response; PR, partial response; SD, stable disease; PD, disease progression; Response group, the first-time evaluation results was CR or PR; No-Response group, the first-time evaluation results was SD or PD; OR, odds ratio; CI, confidence interval; ADC, apparent diffusion coefficient; D, slow diffusion coefficient; D^*^, fast diffusion coefficient; f, perfusion-related diffusion fraction.

### Development and Performance of the Nomogram

**Figure 4:**
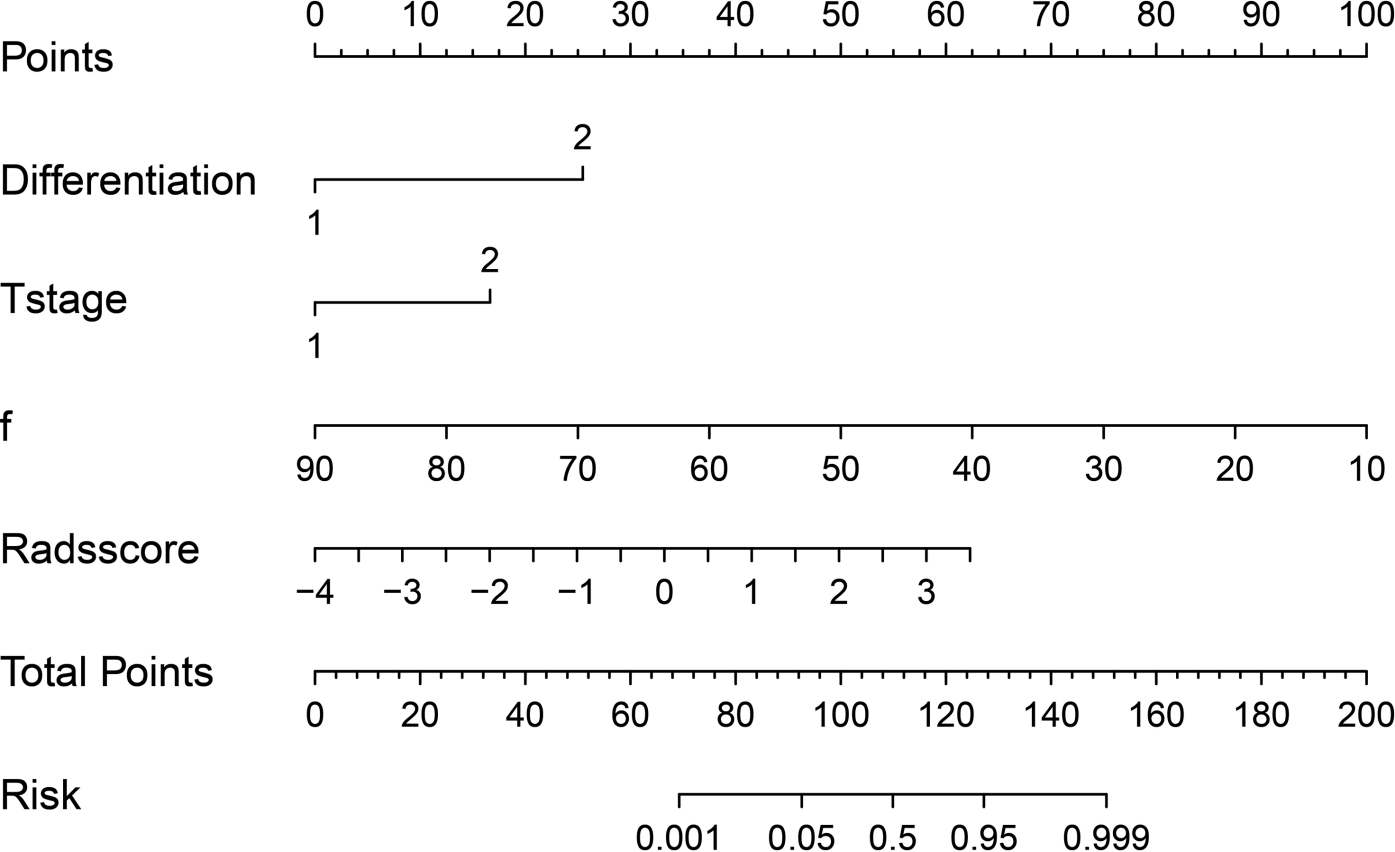
A regression coefficient-based nomogram to predict the possibility of nCRT resistant in LARC patients. f, perfusion-related diffusion fraction.

**Figure 5:**
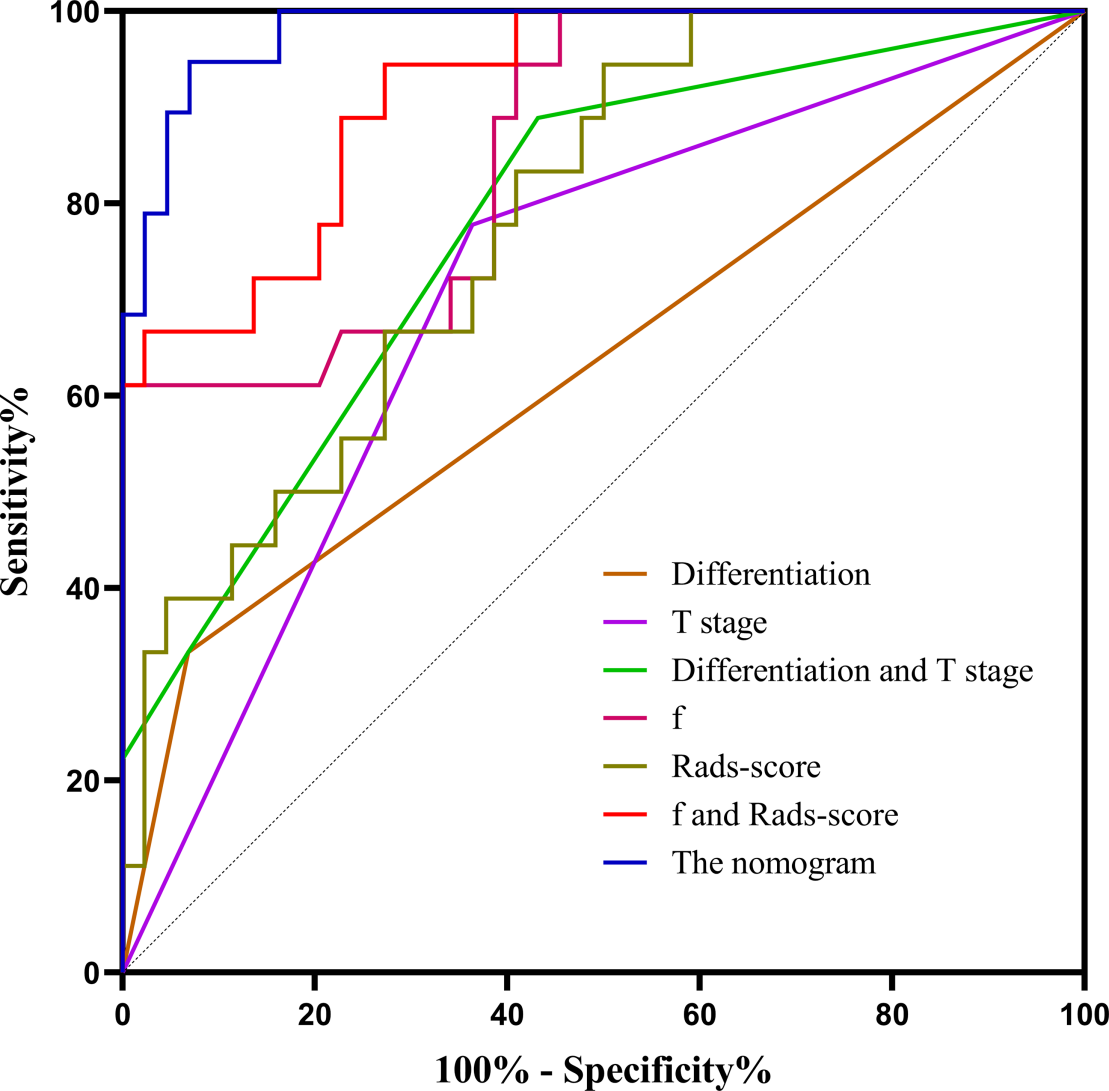
The ROC curves of different parameters and models. f, perfusion-related diffusion fraction; Rads-score, radiomics score.

**Figure 6:**
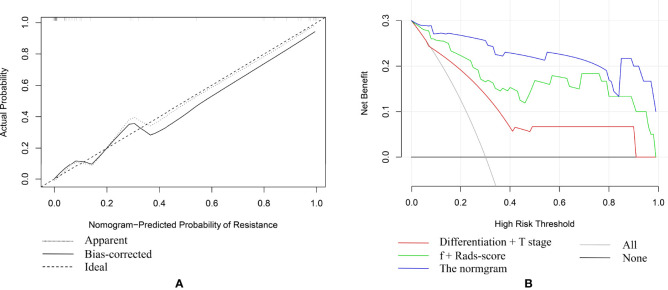
**(A)** The fivefold cross-validation plot of the nomogram. **(B)** Decision curve analysis (DCA) of different models.

Based on combination of the differentiation, T stage, f value and Rads-score, a nomogram was created to predict tumor resistance to nCRT in patients with LARC (**Figure 4**). AUCs of differentiation, T stage, differentiation and T stage, f, Rads-score, f and Rads-score and the nomogram were 0.633, 0.707, 0.778, 0.857, 0.783, 0.917, 0.979, respectively (**Figure 5**) (**Table 5**). The nomogram calibration curve revealed that the observation and prediction were conducted in a good agreement (**Figure 6A**). C-index was 0.979, and Brier score was 0.052. Decision curve analysis (DCA) showed that the model developed by combining the differentiation, T stage, f value and Rads-score had the largest net benefit (**Figure 6B**).

**Table 5:** The predictive performance of the model in rectal cancer patients.

Parameters	AUC	Sensitivity (%)	Specificity (%)	Accuracy (%)
Differentiation	0.633	0.333	0.932	0.774
T stage	0.707	0.778	0.636	0.661
Differentiation and T stage	0.778	0.889	0.568	0.661
f	0.857	1.000	0.611	0.887
Rads-score	0.783	0.944	0.500	0.629
f and Rads-score	0.917	0.944	0.727	0.790
The nomogram	0.979	0.944	0.909	0.919

f, perfusion-related diffusion fraction; Rads-score, radiomics score.

### Internal Validation of the Nomogram Model

To assess model’s discrimination and prediction ability, the nomogram model was internally validated using ten-fold cross-validation. The results showed that the average AUC value was 0.967, and the average C-index was 0.966.

## Discussion

In this study, it was found that the differentiation, T stage, f value and Rads-score were related to tumor resistance to nCRT in patients with LARC. We developed and validated a nomogram which incorporated the differentiation, T stage, f value and Rads-score. The results showed that the nomogram could yield the highest AUC. Calibration curve of the nomogram revealed that the observation and prediction were in a good agreement. DCA indicated that the nomogram model had the largest net benefit.

Since the introduction of quantitative imaging, an increasing number of studies on quantitative imaging have been conducted to improve the diagnosis and treatment of cancer and many of these studies had demonstrated the widespread use of quantitative imaging in the clinical setting (28, 29). For instance, Ren et al. established an MRI-based radiomic signature from combined contrast-enhanced T1-weighted and T2-weighted images to predict the preoperative staging of head and neck cancers (30). Zheng et al. combined the IVIM-DWI parameters (D, f) and texture features (GLCM-correlation, GLRLM-LRE, and GLSZM-ZE) to establish a nomogram for early treatment response prediction in patients with cervical cancer after concurrent chemoradiotherapy (AUC=0.975) (31). Jia et al. performed IVIM-DWI measurement and MRI radiomics feature extraction on 123 rectal adenocarcinoma patients and established a nomogram. The nomogram model of D* and f values combined with Rads-score achieved a good performance in assessing non-enlarged lymph node metastasis of rectal adenocarcinoma preoperatively (AUC=0.864) (26). In this study, we combined the differentiation, T stage, f value and Rads-score to establish a nomogram and our nomogram showed good performance in predicting treatment response to nCRT in LARC patients (AUC=0.967).

This study found that poor differentiation and high T stage were associated with poor response to nCRT in patients with LARC, and these two factors were also independent predictors of tumor resistance to nCRT. Therefore, we established a predictive model (model 1) which combined the differentiation and T stage. The AUC of the model was 0.778, suggesting that the model had a certain predictive value. In a previous study, Huang et al. found that patients in the poorly differentiated group had a worse prognosis than the non-poorly differentiated group who received nCRT for rectal cancer treatment (32). Xu et al. found that in T2N0M0 colorectal cancer patients, the well-differentiated group had a better prognosis than the poorly differentiated group, and the differentiation was an independent prognostic factor in T2N0M0 colorectal cancer (33). The poor differentiation has been shown to be associated with bowel penetration, lymph node involvement and vascular invasion, suggesting that it is a risk factor for colorectal cancer invasion and dissemination (34). T stage reflects the depth of tumor infiltration and correlates with cancer invasion, and several studies have explored the prognostic value of T stage in the colorectum (35–37). It was found that the T4 stage was an independent risk factor for tumor resistance to nCRT in LARC. In a retrospective study, Xu et al. found that the T4 stage was an independent risk factor for shorter overall survival (OS) in patients with colorectal cancer (38). Chen et al. evaluated several microscopic features of stage T4 cancers and suggested that tumors that penetrate the visceral peritoneum and directly invade other organs or structures through malignant invasion were associated with poor survival (39). In summary, the poor differentiation and high T stage of rectal cancer may reflect the high degree of malignancy, which may lead to tumor resistance to nCRT and poor efficacy.

In addition to clinicopathological parameters, this study also found that low f value and high Rads-score were significantly associated with poor efficacy. We combined f and Rads-score to establish the second prediction model (model 2) of tumor resistance to nCRT in LARC and the model had a good predictive performance, AUC=0.917. The f value represents the voxel’s blood volume fraction, which can reflect the capillary density and is related to the blood perfusion in the tissue (40). A previous study had shown that a higher f value may reflect a stronger blood supply to the tumor, which can deliver more chemotherapeutic medications to the tumor tissue, promoting tumor regression (41). Hu et al. found that in rectal cancer patients who received nCRT, the f value of the pCR group was significantly higher than that of the non-pCR group (42). Bakke et al. found that IVIM-DWI could be used to clarify the degree of histopathological regression of LARC after nCRT, and patients with high pre-treatment f value had better tumor regression (43). These studies confirmed that LARC patients with high pre-treatment f value had a better response to nCRT.

TA is a quantitative image post-processing technique, which can objectively reflect the underlying biological characteristics and heterogeneity of tumors due to its quantitative extraction and analysis of the pixel distribution of the lesion area (44). In a previous study, Lu et al. found that High DISS (gray-level run-length matrix_Dissimilarity) on sagittal fat-suppression T2WI and high DISS and ENTR (gray-level co-occurrence matrix_Entropy) on transverse T2WI could predict high T stage in rectal cancer (45). In a study of TA for early prediction of patients’ response to nCRT in LARC, Park et al. found that GLRLM_LRLGE (gray-level run-length matrix_Long Run Low Gray Level Emphasis) on T2WI images could predict tumor recurrence after treatment (46). In this study, the texture parameters of DWI and T2WI images were analyzed and we filter out these two parameters [glrlm_LongRunEmphasis (T2WI) and firstorder_Median (IVIM)] through texture features and calculated the Rads-score, both of which were found to be positively correlated with Rads-score. Multivariate results showed that Rads-score was an independent risk factor for tumor resistance to nCRT in LARC. The glrlm_LongRunEmphasis is a measure of long run length distribution, with a higher value indicating longer run lengths and coarser structural textures. The firstorder_Median is the ROI’s median gray level intensity. These two parameters can represent heterogeneity and subtle changes within the tumor. The results of this study showed that the rough texture and gray intensity in the RIO were more resistant to nCRT.

Most previous studies on rectal cancer only explored the role of IVIM-DWI or texture parameters of a single sequence image (47–49). In this study, two sequence image texture parameters were analyzed and combined with clinicopathology, IVIM-DWI and texture parameters to establish a nomogram for predicting tumor resistance to nCRT in LARC, thus richer and more accurate than the results obtained in previous studies. Compared with model 1 and model 2, the nomogram had higher predictive power and larger net benefit. The nomogram established a risk prediction system for tumor resistance to nCRT in LARC, so that appropriate treatment can be formulated for different patients, which is beneficial to patient recovery.

There are several limitations of this study. First, the manual image segmentation process might be affected by some subjective and objective factors. Second, some cases with small lesions were excluded due to the need for sufficient pixels to ensure the reliability of IVIM-DWI and TA parameter measurements, therefore, the results we obtained might be biased. Third, this study lacks validation with an external cohort. Finally, the present study was a single-center retrospective study with small sample size. Thus, it is necessary to conduct a multi-center prospective study with a large sample size for verification in the future.

## Conclusions

In conclusion, the differentiation, T stage, f value and Rads-score were independent predictors of tumor resistance to nCRT in LARC. The nomogram model combining the differentiation, T stage, f value and Rads-score showed promising performance in estimating the risk of tumor resistance to nCRT in patients with LARC.

## Data Availability Statement

The raw data supporting the conclusions of this article will be made available by the authors, without undue reservation.

## Ethics Statement

The studies involving human participants were reviewed and approved by Anhui Provincial Hospital Ethics Committee. Written informed consent for participation was not required for this study in accordance with the national legislation and the institutional requirements.

## Author Contributions

RS, SW, YH, FG, ML and WC: conception and design. RS, SW, HS, YC, JZ and HJ: collection and arrangement of data. RS, SW, YH, FG and YZ: data analysis and manuscript writing. All authors contributed to the article and approved the submitted version.

## Funding

This work was supported by Natural Science Foundation of Anhui Province (No. 1808085MH234 and 1408085MH179), Anhui Province Key Research and Development Program Project (No. 202104j07020044), Health Commission of Anhui Province Scientific Research Project (No. AHWJ2021b105) and Hefei Key Common Technology Research and Major Scientific and Technological Achievement Project (No. 2021YL005).

## Conflict of Interest

The authors declare that the research was conducted in the absence of any commercial or financial relationships that could be construed as a potential conflict of interest.

## Publisher’s Note

All claims expressed in this article are solely those of the authors and do not necessarily represent those of their affiliated organizations, or those of the publisher, the editors and the reviewers. Any product that may be evaluated in this article, or claim that may be made by its manufacturer, is not guaranteed or endorsed by the publisher.
